# *Aedes albopictus* host odor preference does not drive observed variation in feeding patterns across field populations

**DOI:** 10.1038/s41598-022-26591-3

**Published:** 2023-01-04

**Authors:** Kara Fikrig, Noah Rose, Nathan Burkett-Cadena, Basile Kamgang, Paul T. Leisnham, Jamie Mangan, Alongkot Ponlawat, Sarah E. Rothman, Tanise Stenn, Carolyn S. McBride, Laura C. Harrington

**Affiliations:** 1 Cornell University, Ithaca, NY USA; 2 Princeton University, Princeton, NJ USA; 3 University of Florida, Vero Beach, FL USA; 4 Centre for Research in Infectious Diseases, Yaoundé, Cameroon; 5 University of Maryland, College Park, MD USA; 6 Armed Forces Research Institute of Medical Sciences (AFRIMS), Bangkok, Thailand

**Keywords:** Animal behaviour, Entomology

## Abstract

Laboratory and field-based studies of the invasive mosquito *Aedes albopictus* demonstrate its competency to transmit over twenty different pathogens linked to a broad range of vertebrate hosts. The vectorial capacity of *Ae. albopictus* to transmit these pathogens remains unclear, partly due to knowledge gaps regarding its feeding behavior. Blood meal analyses from field-captured specimens have shown vastly different feeding patterns, with a wide range of anthropophagy (human feeding) and host diversity. To address this knowledge gap, we asked whether differences in innate host preference may drive observed variation in *Ae. albopictus* feeding patterns in nature. Low generation colonies (F2–F4) were established with field-collected mosquitoes from three populations with high reported anthropophagy (Thailand, Cameroon, and Florida, USA) and three populations in the United States with low reported anthropophagy (New York, Maryland, and Virginia). The preference of these *Ae. albopictus* colonies for human *versus* non-human animal odor was assessed in a dual-port olfactometer along with control *Ae. aegypti* colonies already known to show divergent behavior in this assay. All *Ae. albopictus* colonies were less likely (p < 0.05) to choose the human-baited port than the anthropophilic *Ae. aegypti* control, instead behaving similarly to zoophilic *Ae. aegypti*. Our results suggest that variation in reported *Ae. albopictus* feeding patterns are not driven by differences in innate host preference, but may result from differences in host availability. This work is the first to compare *Ae. albopictus* and *Ae. aegypti* host preference directly and provides insight into differential vectorial capacity and human feeding risk.

## Introduction

Mosquito blood feeding behavior is a critical determinant of pathogen transmission. Some species have innate host preferences, actively choosing to feed on one host species or class over others^1–3^. Other mosquito species are host generalists, and exhibit little to no preference for particular host species or groups. Host preference plays a role in host contact rates, interacting with external factors, such as host availability, to influence feeding patterns in the field^1,4,5^. Feeding patterns, in turn, influence the probability of mosquito contact with infectious host reservoirs and onward transmission to susceptible hosts^6,7^.

Despite the important role of host preference in pathogen transmission, this trait is not well characterized for the tiger mosquito, *Aedes albopictus*, a highly invasive nuisance species with the potential to transmit over twenty pathogens that infect a range of vertebrate host species^8,9^. Several of these pathogens share another mosquito vector, *Ae. aegypti*, including dengue, Zika, and chikungunya viruses. In contrast to *Ae. albopictus*, *Ae. aegypti* host preference is well characterized. In its invasive range outside of Africa, *Ae. aegypti* is strongly anthropophilic, preferring the odor of humans over that of other host species^10,11^. Within its ancestral range in Africa, *Ae. aegypti* is more diverse and exhibits a range of host preferences, from zoophilic (non-human preferring) to anthropophilic (human preferring)^11–13^. Human-preferring populations in and out of Africa are genetically related, sharing common mutations in several chromosomal regions^11^, which provides additional support for the genetic underpinnings of human preference^13^.

*Aedes albopictus* host preference has only been assessed twice to date, despite its importance as a major vector and nuisance species. In Thailand, landing catches were performed, comparing attraction of wild mosquitoes to human, pig, buffalo, dog, and chicken^14^. In La Réunion, preference assays were conducted with human, cow, dog, goat, and chicken, measuring choice between human and each of the non-human animals by releasing mosquitoes in an enclosure and subsequently assessing host feeding rates^15^. The results of both studies indicate a preference for humans over the other animals tested. However, the results from the La Réunion study may have been influenced by host defenses^15^. Human subjects may have avoided defending themselves since they knew they were in a scientific study while the non-human animals would likely have exhibited typical host defense behaviors that may have impacted feeding success^16^. Note, however, host defenses were not addressed in the text^15^. In La Réunion, no-choice (single host) assays were also conducted, measuring feeding on human, pig, goat, cow, dog, duck, chicken, rat, chameleon, gecko, mouse, and shrew in the absence of other hosts^15^. *Aedes albopictus* fed readily on chicken, human, dog, and cow, and significantly less often on all other hosts assessed.

While little is known about *Ae. albopictus* host preference, there has been robust investigation of its feeding patterns. Feeding patterns are distinct from host preference in that patterns describe mosquito-host associations in nature and are influenced by environmental and biological parameters, whereas preference describes the innate tendency of a mosquito species to choose a certain host over others^1^. *Aedes albopictus* feeding patterns have been assessed across nineteen blood meal analysis studies from across the world, the results of which exhibit a remarkably diverse range of feeding (reviewed by Fikrig and Harrington, 2021^1^). The percent of blood meals identified as human ranged from 3.9 to 100% and the number of host species identified ranged from three to fifteen. The cause of this striking variability in feeding patterns is unknown. Methodological differences, such as blood meal analysis and collection techniques may explain some of the differences. There were also likely differences in host availability. However, only three of these studies quantified host availability^17–19^, so it is impossible to retrospectively determine the extent to which external factors drove the differences in host usage. Another possibility is that *Ae. albopictus* populations vary in genetically-based host preference, similar to *Ae. aegypti* populations within Africa, thus driving divergent feeding patterns^11^.

*Aedes albopictus* has high levels of phenotypic variation for numerous traits, including diapause^20^, fecundity-size relationships^21^, competitive interactions^21^, larval growth rate^22^, and viral susceptibility^23^. It also has a large genome^24^ and substantial levels of genetic variation^25–27^, although the level of variation among populations is different in different parts of the world^28^. *Aedes albopictus* genetic variation has been shown to underpin phenotypic variation of several traits, including vector competence^29^ and diapause^30–32^. This trait variation has been credited for the impressive invasive potential of *Ae. albopictus* and its widespread establishment across a variety of climates and habitats^33,34^.

It is unclear whether such variation exists for host preference among *Ae. albopictus* populations; the two host preference studies conducted to date do not provide insight into the possibility that innate host preferences vary in relation to observed differences in feeding patterns. The first *Ae. albopictus* host preference study referenced above was conducted in Thailand^14^, where a blood meal analysis revealed that 100% of blood fed *Ae. albopictus* fed on humans, the most anthropophagic feeding pattern reported to date^35^. The other was conducted in La Réunion, which was the site of a chikungunya epidemic transmitted by *Ae. albopictus*, suggesting a high level of human feeding (although no blood-feeding pattern study has been performed to corroborate this)^36,37^. There has been no experimental assessment of host preference from locations where *Ae. albopictus* populations have lower levels of anthropophagy (human feeding), nor any comparison of host preference between discrete populations.

We investigated whether population-level variation in *Ae. albopictus* host preference drives the divergent feeding patterns reported around the world. Using a dual-port olfactometer to simultaneously present human and guinea pig odors, we measured the host preference of low-generation *Ae. albopictus* colonies derived from six populations around the world: three from populations with previously reported low levels of anthropophagy in the United States (New York^17^, Maryland^38^, and Virginia^39^), and three from populations with high levels of anthropophagy (Florida, USA^40^, Cameroon^41^, and Thailand^35^). We directly compared these colonies to anthropophilic and zoophilic *Ae. aegypti* colonies, thus also providing the first direct comparison of the host preferences of these two vector species.

## Results

Using a dual-port olfactometer, we measured the host preference of *Ae. albopictus* colonies derived from three anthropophagic and three zoophagic populations, as well as one zoophilic and two anthropophilic *Ae. aegypti* colonies. Eight replicates were conducted across two separate experimental rounds. Additionally, in the second experimental round, we assessed biological replicate colonies of three *Ae. albopictus* populations (established from a site at least 1.5 km away from the primary colony collection site) and an anthropophilic *Ae. aegypti* transport control (to control for potential effects of transportation from the laboratory at Cornell to Princeton). For each of the colonies, we report the predicted probability of choosing human over guinea pig. We assessed whether differences in host choice existed between the colonies by analyzing the data together and for each experimental round separately.

**Figure 1:**
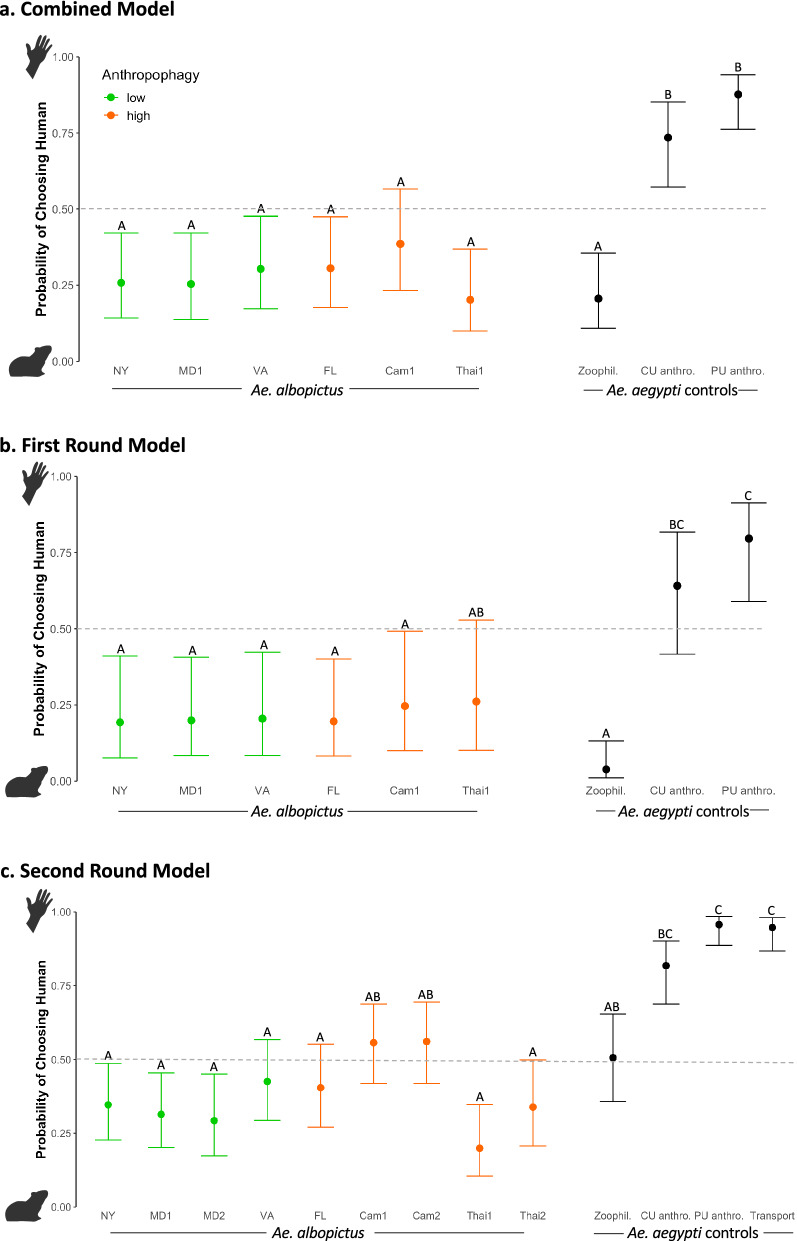
This figure shows the predicted probability of choosing human for each of the colonies in: (**a**) the combined model, (**b**) the first round model, and (**c**) the second round model. Within each graph, colonies that do not share a letter above the upper confidence limit are statistically different (p < 0.05). The green colonies are *Ae. albopictus* derived from populations with previously reported low levels of anthropophagy, the orange colonies are *Ae. albopictus* from populations with previously reported high levels of anthropophagy, and the black are *Ae. aegypti* control colonies. The dashed grey line indicates a 0.5 probability of choosing human, the level at which a colony would be equally likely to choose human or guinea pig; above this line, the colony is more likely to choose human and below, guinea pig. The first and second round trials were performed with minor methodological differences, including arm presentation. The following colony abbreviations were used: *NY* New York, *MD1* Maryland 1, *VA* Virginia, *FL* Florida, *Cam1* Cameroon1, *Thai1* Thailand 1, *Zoophil.* Zoophilic, *CU anthro.* Cornell anthropophilic, *PU anthro.* Princeton anthropophilic.

For the combined analysis, all six *Ae. albopictus* colonies were more likely to choose guinea pig than human. The predicted probabilities of choosing human were below 0.5, including both the anthropophagic and zoophagic *Ae. albopictus* colonies (Fig. 1A, Supplemental Table S1). As expected, the zoophilic *Ae. aegypti* colony was more likely to choose guinea pig than human and the two anthropophilic *Ae. aegypti* colonies were more likely to choose human than guinea pig. All *Ae. albopictus* colonies were clearly zoophilic, with the exception of Cameroon 1. This was the only *Ae. albopictus* colony with an upper confidence limit that crossed 0.5, the dividing line between whether a colony is more likely to choose human or guinea pig (predicted probability = 0.386, lower CL = 0.232, upper CL = 0.566). Because this upper confidence limit was greater than 0.5, it is the only *Ae. albopictus* colony for which we cannot preclude the possibility of human preference under these experimental conditions, although the predicted probability was below 0.5. Notably, Cameroon 1, and all five other *Ae. albopictus* colonies were still significantly less likely to choose human than the two anthropophilic *Ae. aegypti* colonies (α = 0.05) and did not behave significantly differently from the zoophilic *Ae. aegypti* (Supplemental Table S1).

We then examined the results for each round separately due to slight technical differences between the method of human host presentation (elbow versus forearm and hand). We also added a transport control and biological replicates for three *Ae. albopictus* colonies in the second round to control for potential effects of transportation from the laboratory at Cornell to Princeton (ca. 4-h drive in a car with human odor) and potential founder effects, respectively. The first-round model included data from the four first-round replicates and was largely consistent with our analysis of both rounds combined with slight differences in the probability of choosing human over guinea pig (Fig. 1B, Supplemental Table S1). One notable difference in the first round alone compared with the combined model is that the Thai *Ae. albopictus* did not present a significantly different probability of choosing human compared with the Cornell anthropophilic *Ae. aegypti* colony (p = 0.103), although it remained significantly different from the Princeton anthropophilic *Ae. aegypti* colony (p = 0.007). All other significant and non-significant relationships remained the same as the combined model (α = 0.05). In this round, the colonies exhibited a spectrum of response rates (percent of released mosquitoes that entered a host port), ranging from 11.2 to 25.0% for the *Ae. albopictus* colonies and between 41.6% and 71.3% for the *Ae. aegypti* colonies.

Results of the second round, which included the remaining four replicates, were also consistent with the combined model (Fig. 1C, Supplemental Table S1). All nine *Ae. albopictus* colonies (the six original colonies and three biological replicates) were not significantly different from one another (p > 0.05). The transport control (the Princeton anthropophilic *Ae. aegypti* colony reared at Cornell) did not behave differently from the same colony raised at Princeton (p = 1.00), suggesting that preference behavior was not modified by the transport of mosquitoes from Cornell to Princeton and slight differences in rearing. The two Cameroonian colonies (p = 0.260 and p = 0.2434) and the zoophilic *Ae. aegypti* colony (p = 0.122) were not different from the Cornell anthropophilic *Ae. aegypti*, but were different from the Princeton anthropophilic *Ae. aegypti,* as were all other *Ae. albopictus* colonies (p < 0.01)*.* All other significant and non-significant relationships remained the same as the combined model (α = 0.05). In this round, the colony response rates ranged from 16.5 to 39.3% for the *Ae. albopictus* colonies and between 24.3% and 45.8% for the *Ae. aegypti* colonies.

### Variation by experimental subject

**Figure 2:**
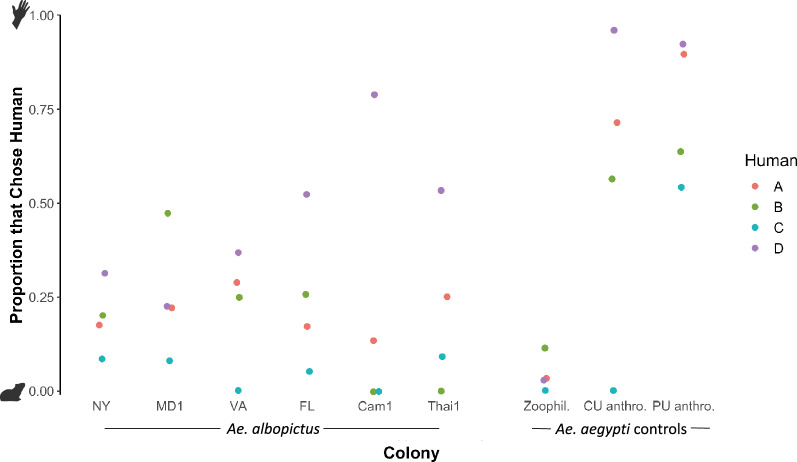
This figure shows the proportion of host-seeking mosquitoes that chose human over guinea pig for each of the colonies. Each point represents the results of one replicate in the first round, with each color representing one of the four human subjects used in this round.

Individual host variation in mosquito attraction is a well-documented phenomenon^42^. To account for this, we examined mosquito behavioral responses across the individual experimental subjects (human and guinea pig). In the first round, a different human subject was used for each of the four replicates (Fig. 2). We detected significant differences in the predicted probability of choosing human between four of the six paired comparisons (p < 0.05) and one pair with a marginally significant difference (p = 0.0514) (GLMM, human = fixed effect, colony = random effect).

**Figure 3:**
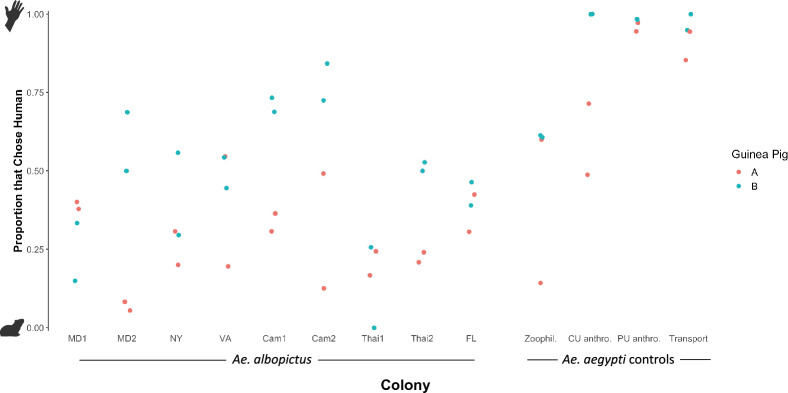
Scatter plot of the proportion of host-seeking mosquito that chose human over guinea pig for each of the colonies, with the color representing the guinea pig. Each point represents the results of one replicate in the second round.

In the second round, the same human was used for all four replicates and two guinea pigs were used for two replicates each. This experimental set up allowed us to isolate the effect of guinea pig; one guinea pig was more attractive than the other (p < 0.0001; Fig. 3) (GLMM, guinea pig = fixed effect, colony = fixed effect). When stratified by guinea pig, the pairwise estimated marginal mean comparisons between colonies for each guinea pig did not change the main conclusion that the *Ae. albopictus* colonies were less likely to choose human than anthropophilic *Ae. aegypti* and behaved similarly to zoophilic *Ae. aegypti.* Although two guinea pigs were also used for two replicates each in the first round, each human was only paired with one guinea pig due to animal use constraints, making it difficult to isolate the effect of guinea pig, resulting in no significant difference between the two guinea pigs in the first round or combined models (p = 0.065 and p = 0.063, respectively).

## Discussion

This study is the first to compare the host preference of *Ae. albopictus* across multiple populations, the first to characterize host preference of zoophagic *Ae. albopictus* populations, the first to assess *Ae. albopictus* host odor preference separately from other cues in a controlled laboratory setting, and the first to directly compare host preference of *Ae. albopictus* to that of *Ae. aegypti.* Our results provide important insight into the behavior of this mosquito species and can help us to evaluate the relative importance of these vector species for transmission of anthroponotic and zoonotic pathogens.

Our results do not support the hypothesis that differences in *Ae. albopictus* host preferences drive observed differences in feeding patterns among populations in nature. The three anthropophagic *Ae. albopictus* populations we tested were not more likely to choose a human host *versus* a guinea pig compared with the three zoophagic populations. Further, we found no significant differences between any of the *Ae. albopictus* populations tested, representing a broad global distribution, including several populations from across the Eastern United States, Asia and Africa. Our results contrast with findings for two other species, including variation in *Ae. aegypti* host preference within Africa and between African and non-African populations, measured using the same experimental design we used in our study^11^, and *Culex annulorostris* within Australia^43^. At a finer geographic scale, we also did not detect variation for host preference in the locations where we collected paired biological replicate colonies of *Ae. albopictus* within about 1.5 km of one another, similar to the absence of fine scale variation between paired *Ae. aegypti* colonies collected within 5–10 km of each other^11^. Given the wide distribution of *Ae. albopictus* around the world, it is possible that host preference variation exists at locations that were not included in this study. The colonies tested in our study do not encompass all genetic backgrounds identified globally^44^.

All *Ae. albopictus* we tested, regardless of geographic origin and previously reported feeding patterns, were significantly less likely to choose human over guinea pig than the previously characterized Princeton anthropophilic *Ae. aegypti* from Thailand and none were significantly different from the previously characterized zoophilic *Ae. aegypti* from Uganda^11^. Human preference responses between our two anthropophilic *Ae. aegypti* colonies varied but were not significantly different.

Our results suggest that *Ae. albopictus* host preference behavior is closer to that of ancestral *Ae. aegypti* populations than to that of the invasive *Ae. aegypti* lineage that evolved to specialize in biting humans and then spread out of Africa and around the world. We can therefore conclude that *Ae. albopictus* is less anthropophilic than invasive *Ae. aegypti.* In the past, *Ae. albopictus* has been considered both an anthropophilic and a generalist blood feeder^1^; however *Ae. albopictus* has typically been presumed to be less anthropophilic than *Ae. aegypti* (e.g.^45^). The quantification of their relative anthropophily in this study has implications for understanding the relative threat posed by the two species, which transmit many of the same pathogens (e.g. dengue, Zika, and chikungunya) and live in similar habitats with overlapping distributions^46^. *Aedes albopictus* may only pose a comparable threat for transmission of anthroponoses in settings where humans are highly available, such as densely populated urban areas with open housing structures. It also suggests that in settings where humans are available at intermediate levels alongside other hosts, *Ae. albopictus* is more likely to transmit zoonotic pathogens than *Ae. aegypti*.

Our assays also demonstrated variation in preference for host individuals of the same species (both individual humans and individual guinea pigs). It is well-established that there is variation in mosquito attraction to different humans (reviewed by Martinez et al., 2021^42^). It is notable that variation in human preference was observed in the first round of our experiment despite the recruitment process to select humans with a relatively high level of attractiveness to anthropophilic *Ae. aegypti*, which may have been expected to limit the observed variation in attraction among human hosts. We do not know whether *Ae. aegypti* and *Ae. albopictus* prefer the same individual hosts. It is possible that *Ae. albopictus* responds to different odor components than *Ae. aegypti,* which would mean that human subjects chosen to maximize the response of anthropophilic *Ae. aegypti* (see Methods) might create or increase the perceived difference in human preference between the two species in the first experimental round. Mosquito species can respond differently to bacterial volatiles from different host species^47^, which may also be the case for differences between individuals of a given host species.

Variation in intra-species attraction among non-human hosts has been reported previously, with attraction varying by physiological stage in rats^48^, stress hormones in zebra finches^49^, and body mass and metabolic rate in house sparrows^50^. The two guinea pig hosts used in our study were both mature females, however they were different ages, with the older of the two being the more attractive. We did not measure body mass, metabolic rate, stress hormones, or any other parameter that may have driven the difference in attraction of the two guinea pigs. Additional experiments designed to assess attraction to individual hosts may provide more insight on variation in attractiveness among individual humans and among individual non-human hosts. Many host preference studies use one or a few individuals to represent the human and non-human host species; it should be acknowledged that the individuals chosen for each species may impact the magnitude and even directionality of the measured preference.

**Figure 4:**
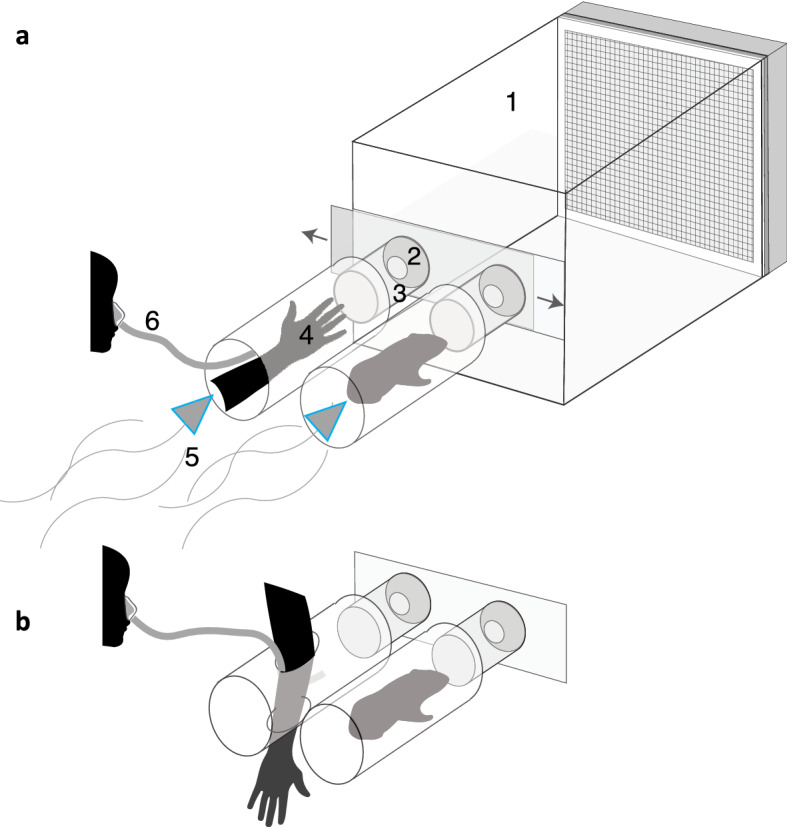
(**a**) The dual-port olfactometer consists of the following components: 1. the mosquito chamber, in which mosquitoes are released and allowed 10 min to acclimate prior to odor exposure; 2. funnel that allows mosquito entry into ports after the sliding door is opened but limits return of mosquitoes from the trap to the mosquito chamber; 3. mosquito trap, with a funnel on one end and a screen on the other to prevent access to hosts; 4. host chambers that hold the host for odor presentation; the method of human arm presentation pictured here was used in the second experimental round (the full forearm and hand were inserted through the far end of the tube and exposed to air flow, with the breathing tube inserted alongside the arm). An opaque panel (not pictured) divided the hosts from the mosquitoes such that they were not visible from the mosquito chamber prior to host choice; 5. wind source, which was created by blowing a carbon-filtered air source over the hosts in the first round (with a nozzle connected to each host chamber and an air outtake at the other end of the mosquito chamber; ~ 0.6 m/s windspeed), and in the second round, created by a window fan fitted to the far end of the mosquito chamber, producing air flow by sucking air from the room through the host chambers and the mosquito chamber. (**b**) In the first experimental round, the human arm was displayed by inserting the arm perpendicularly through the host chamber, with the hand protruding from the opposite side, such that the elbow was exposed to air flow. The breathing tube (6) was also inserted into the host chamber. Schematics modified from Metz et al. (2022)^53^.

In our study, the way human odor was presented to the mosquitoes differed between round one and two. In round one, air was passed over only the middle section of a human arm, from just below to just above the elbow (Fig. 4). In round two, air was passed over the full forearm and hand. We found that changing the arm display from the elbow to the full forearm and hand could increase the level of attractiveness of an unattractive human subject. Exposing the full forearm and hand may have increased available surface area or exposed different odors emitted by different parts of the arm. Some mosquito species exhibit preference for certain body parts, based on preference for specific microbial volatiles in those areas^51^, although such body part-specific preferences are not always detected^52^. Despite this change in odor presentation, the patterns of host attraction remained similar across the first and second rounds.

In addition to the challenge of individual host variation in attractiveness, laboratory preference assays need to ensure field-relevance of the mosquito colonies. Collection and colony rearing techniques can lead to founder effects, bottlenecks, and selection^54,55^. We attempted to limit selection pressure on host preference by sampling *Ae. albopictus* population egg or larval stages, except for the Thai *Ae. albopictus,* which were collected via human-landing catch, potentially selecting for human preference. Despite this difference, the Thai *Ae. albopictus* colonies were not more likely to choose human than the other colonies when tested in the olfactometer. To avoid laboratory selection for host preference, as has been described in other species^56^, mosquitoes were tested within just a few generations of colony establishment (F2-F4), were fed on artificial feeders with minimal host cues, and were given ample time to blood feed (until > 90% were fed). Therefore, we expect that the laboratory colonies tested in these experiments are representative of the field populations.

The goal of our study was to understand, for the first time, levels of *Ae. albopictus* anthropophily across global geographic isolates. Our experimental design exposed a small but consistent part of human hosts to mosquitoes and was conducted in a controlled laboratory setting. We chose to use guinea pig as the non-human host because it is one of the most common hosts used in *Ae. aegypti* host odor preference assays, allowing us to assess *Ae. albopictus* host preference using a host that elicits replicable, divergent behavior between the *Ae. aegypti* control colonies. Domesticated guinea pigs have been dispersed by trade throughout much of the world from their native origin in South America^57^, resulting in an overlapping geographic distribution with the globally invasive *Ae. albopictus.* Although guinea pig has never been reported in *Ae. albopictus* blood meal contents, it is unknown whether guinea pigs were present at the study sites. As such, it is possible that guinea pigs serve as a natural host in the field, but it has yet to be demonstrated. Further research is needed to understand the probability of choosing a human over more relevant non-human animals in field settings given large variation in host sizes and the need to include a range of hosts naturally available to *Ae. albopictus*. Our results are in contrast to the two previous assessments of *Ae. albopictus* host preference, which tested the full human body versus various non-human animals and concluded that *Ae. albopictus* prefer humans to non-human animals^14,15^. In the latter study, host defenses may have contributed to the conclusion of human preference. Additional *Ae. albopictus* host preference assays should be conducted using different experimental techniques and host animals to better understand this trait and tease out potential artifacts of experimental design.

Here, we demonstrated for the first time that *Ae. albopictus* host preference is not likely to be the driver of the highly variable feeding patterns reported in the literature for this species. The *Ae. albopictus* colonies that we tested were consistently zoophilic in contrast to the highly anthropophilic invasive lineage of *Ae. aegypti,* further supporting our understanding that *Ae. aegypti* has a higher capacity to transmit arboviruses between humans than *Ae. albopictus*.

## Methods

### Colony establishment

Based on previously reported *Ae. albopictus* blood meal analysis studies, three populations with high levels of anthropophagy and three populations with low levels of anthropophagy were selected. The high anthropophagy populations included Cameroon (99.4% anthropophagy, defined as the percent of all identified bloodmeals identified as human)^41^, Thailand (100% anthropophagy, including 5.7% that fed on both a human and non-human animal)^35^, and Florida, USA (91% anthropophagy)^40^. The low anthropophagy populations, all from the eastern United States, included New York (32.2%)^17^, Maryland (13.6%)^38^, and Virginia (7.3%)^39^. Three of these populations (Cameroon, Thailand, and Maryland) included a biological replicate (a second colony from a site at least 1.5 km away from the primary colony collection site). The collections took place at roughly the same sites as the blood meals were collected in previous studies, except for Thailand because sites could not be reached due to COVID travel restrictions (see Supplemental document S1 for site details). However, time gaps did exist between the blood meal and colony collections, ranging from 18 to 2 years (Thailand and Virginia, respectively). Populations may have evolved in the intervening time, potentially reducing the concordance between population traits that produced the feeding patterns and the host preferences observed here.

Most collections were conducted with oviposition traps, black buckets treated with an attractant (water infused with dog food, hay, or other organic material, depending on site) and lined with paper towel or seed germination paper to collect eggs. At least ten traps were distributed at least 100 m apart, except for the Florida site, which was in a scrapyard that was not sufficiently large for such distant spacing. Collections were conducted over 2–3 weeks and egg sheets were removed multiple times per week. The collections that were not conducted with oviposition traps included Thailand, Cameroon, and one of the Maryland biological replicates (Maryland 2). In Thailand, collections were conducted with human landing catches, which were performed prior to other collections and could not be repeated with the standardized collection methods (designed to avoid selection on host preference) due to COVID travel restrictions. In Cameroon and the Maryland biological replicate, larval collections were conducted. Larvae were collected from at least 10 containers at least 100 m apart.

The control colonies were established prior to this study. The zoophilic *Ae. aegypti* was originally collected in Uganda (ZIK in Rose et al. 2020)^11^, and both the Princeton (T51 in Rose et al. 2020)^11^ and Cornell^58^ anthropophilic *Ae. aegypti* were originally collected in Thailand.

### Mosquito rearing

In the case of oviposition collections, egg sheets were sent directly to Cornell University, where they were maintained in an environmental chamber until hatching (28 °C, 71.9% ± 9.5% relative humidity, 10 h light, 10 h dark, and 2 h dusk/dawn). Egg sheets were soaked in water for 20 min and then vacuum-hatched and provided with a pinch of pulverized fish food (medium Cichlid Gold™ fish food pellets; Hikari, Himeji, Japan). Within 24 h, larvae were transferred from the hatch flask to rearing trays, with 200 larvae, 1 L of distilled water, and 7 fish food pellets in each. Upon pupation, mosquitoes were transferred to cups and placed in cages. Adult females were blood fed using an artificial feeding system, with human blood (Valley Biomedical, Winchester, VA, USA), with sausage casing as the piercing membrane (DCW Casing LLC, Mount Vernon, NY, USA). Colonies were fed at approximately 7, 14, and 21 days post-eclosion. To avoid selection during the feeding process, colonies were monitored to ensure high rates of feeding (> 90%) and were fed a second day if sufficient feeding levels were not reached. Three days post-feeding, oviposition cups were placed into the cage, treated with strained larval water to induce improved egg-laying^59^ and dirt in the case of the Ugandan colony. Three days later, oviposition cups were removed and egg sheets were dried until slightly damp and then maintained in the environmental chamber. All colonies derived from oviposition traps were maintained in colony in this form until they were used in the preference assays in generation F2, except for the primary Maryland colony, which was F3 in the second experimental round.

In Thailand, the adult female mosquitoes captured through human landing catch were brought to the lab and blood fed using human blood via an artificial feeder. The egg sheets derived from these feedings were sent to Cornell University (F1). The Thai *Ae. albopictus* used in experiments were generation F3. In Cameroon, the collected larvae were brought to the lab, reared, and blood fed using rabbit blood (live and via artificial feeder). They were maintained in colony in Cameroon for one more generation and F2 eggs were sent to Cornell University. The Cameroonian *Ae. albopictus* used in experiments were generation F3 and F4. For the second biological replicate from Maryland, larvae were shipped in water to Cornell University. The *Ae. albopictus* from the Maryland biological replicate colony was generation F2 in the experiments. In all cases, upon arrival at Cornell University, the colonies were maintained as described above until preference assays were performed.

The zoophilic *Ae. aegypti* eggs were sent to Cornell from a colony at Princeton and reared as described above. The Cornell anthropophilic *Ae. aegypti* were acquired directly from eggs derived from the Cornell colony, which is maintained similarly to the methods described above, except for blood feeding, which is typically conducted with a live human and periodically with a live chicken. Mosquitoes reared for experiments were reared in the same fashion as the *Ae. albopictus* colonies. The Princeton anthropophilic *Ae. aegypti* were reared at Princeton (F14), with slight differences in rearing protocol: eggs were hatched in deoxygenated water and fed Tetramin Tropical Tablets fish food (Spectrum Brands, Inc) ad libitum until pupation, then transferred to cages. In the second experimental round, the Princeton colony was reared at Cornell as well as Princeton as a transport control—eggs were brought to Cornell and reared alongside the other colonies.

At about five days post-eclosion, colonies were transported to Princeton in a heated car for preference assays (except for the Princeton *Ae. aegypti*, which were already located there). No mortality was noticed. The colonies were given approximately 50 h to acclimate in the rearing chamber at 28 °C (71.9% ± 9.5% RH) before preference assays commenced.

### Preference assays

#### First-round

Preference assays were performed in a dual-port olfactometer (Fig. 4A), using a similar methodology as previous studies^11,13^. Between 50 and 175 females were released in the mosquito chamber and allowed to acclimate for approximately ten minutes. Only the first and second replicate of the two anthropophilic *Ae. aegypti* colonies were conducted with fewer than 98 females; all others included 98–175 females. After acclimation, the ports were opened and the wind source was turned on, moving carbon-filtered air over the hosts and into the mosquito chamber, presenting the mosquitoes with a human and guinea pig odor plume emanating from the respective ports. The human odor source included the elbow of the human, inserted perpendicularly through two holes in the host chamber, and breath, exhaled from the nose every 30 s through a breathing tube (Fig. 4B). The full guinea pig was presented in the other host chamber and allowed to breathe normally. Guinea pig was chosen as the non-human host because it is one of the most common hosts used to assess *Ae. aegypti* host odor preference, eliciting replicable, divergent behavior between the control colonies^11^. The mosquitoes were given ten minutes to fly upwind and enter one of the port traps, where they were prevented from accessing the host via a screen and inhibited from flying back into the mosquito chamber via a funnel. At the conclusion of the ten minutes, the number of mosquitoes that entered each port was counted.

The first round of replicates was conducted over four days. Each day, the six primary *Ae. albopictus* colonies, the zoophilic *Ae. aegypti*, and the two anthropophilic *Ae. aegypti* colonies were tested. The order of testing was rotated so that the colonies would be tested at different times of day. The side on which the human and guinea pig were presented was swapped after approximately half of the replicates were completed each day.

Potential human subjects were tested for attractiveness level prior to inclusion using the two anthropophilic *Ae. aegypti* colonies; the goal of this study was to measure the relative anthropophily of the colonies, so we wanted to include attractive humans to maximize the potential dynamic range of anthropophily observed. We selected four of seven tested human subjects; two were excluded due to low levels of attraction and one due to illness during the first round. Each of the four human subjects were used for one full day of replicates (all nine colonies tested in this round). These participation of humans in olfactometer trials using these methods was approved and monitored by the Princeton University Institutional Review Board (protocol 8170). All participants provided informed consent before participating.

Two guinea pigs were used and rotated after each day of experiments. The guinea pigs periodically defecated or urinated during the trial; when this occurred, the soiled protective sheet at the bottom of both host chambers were removed and replaced with new sheets. The use of guinea pigs in olfactometer trials was approved and monitored by the Princeton University Institutional Animal Care and Use Committee (protocol 1998–20). All methods were carried out in accordance with the corresponding guidelines and regulations.

#### Second round

The second experimental round was performed largely the same as the first round with several exceptions. In the second round, personnel constraints required one human to serve as the human subject for all four replicates, with the added benefit of removing one potential source of variation. However, the human subject in question was deemed relatively unattractive based on the recruitment assays that were conducted prior to the first round and was excluded from that round. Based on anecdotal experience, a pilot was conducted comparing two forms of arm display: the elbow as in the first round (Fig. 4B) versus the full forearm and hand (Fig. 4A). This demonstrated an increase in the probability of choosing human using the full forearm and hand compared with the elbow, which was later confirmed by a small trial (Supplemental document S2). Changing the arm presentation also required changing the airflow system. The arm was inserted through the back of the human host chamber, preventing the connection of the carbon-filtered air system. Instead, the fan attached to the far end of the mosquito chamber was alone responsible for drawing air from the room over the hosts and through the mosquito chamber, as done in a previous study^13^. This may have caused more mixing of host odors; however, efforts were made to reduce this phenomenon: the air exchange in the room holding the dual-port olfactometer is extremely high (multiple exchanges per hour) and the hosts were removed from the room for at least 15 min between each replicate, limiting the accumulation of host odors in the room.

The other difference in the second round was that the three biological replicate colonies and a transport control were included in addition to the nine colonies tested in the previous round. As a result, thirteen colonies were tested each day. The transport control was added to assess whether the transport from Cornell to Princeton impacted the host seeking behavior. The Princeton anthropophilic *Ae. aegypti* were reared at both Princeton and Cornell.

### Data analysis

A Generalized Linear Mixed Model using Template Model Builder (glmmTMB)^60^ with a beta-binomial distribution was employed to evaluate the contribution of several factors to the predicted probability of choosing human for each of the analyses described below (the combined, first round, and second round models that seek to evaluate the effect of colony and the human model, which seeks to evaluate the effect of human). Post hoc analyses were conducted by calculating the estimated marginal means of the effects by using emmeans^61^. A predicted probability of choosing human equivalent to 0.5 indicates no preference between human and guinea pig; between 0.5 and 1 indicates a preference for human and between 0 and 0.5 indicates a preference for guinea pig. Analyses were conducted using R version 4.1.1^62^. Graphs were created using ggplot^63^.

#### Combined model

The data for the six primary *Ae. albopictus* colonies, the zoophilic *Ae. aegypti* and the two anthropophilic *Ae. aegypti* colonies were combined across eight replicates conducted in the first and second experimental rounds, which we refer to as the combined model. In this model, colony, guinea pig, and side of human host chamber were included as fixed effects and with random effects of human and date.

#### First-round model

In this model, colony, guinea pig, and side of human host chamber were included as fixed effects and human as a random effect. Each human was only tested one day in the first round, so date was excluded from this analysis.

#### Second-round model

In this model, colony, guinea pig, and side of human host chamber were included as fixed effects and date as a random effect. Only one human was tested in the second round, so human was excluded from this analysis.

#### Human model

To evaluate the differential attraction to each of the four humans in the first round, human and side of human host chamber were included as fixed effects and colony as a random effect. We did not include guinea pig in this analysis, because each human was only tested against one guinea pig, resulting in nesting of this data.

## Supplementary Information

Supplementary Information 1.

Supplementary Information 2.

Supplementary Information 3.

## Data Availability

Data are deposited in Cornell University Library’s institutional repository, eCommons (https://ecommons.cornell.edu) for preservation and access via the world wide web without restriction at the following https://doi.org/10.7298/k8wf-bh80.
