# Supplementary material for: Aedes albopictus host odor preference does not drive observed variation in feeding patterns across field populations
Source: Sci Rep. 2023 Jan 4;13:130. doi: 10.1038/s41598-022-26591-3 (PMC9813369; doi:10.1038/s41598-022-26591-3)
Supplement: Supplementary file 1 — Supplementary Information 1. [file 41598_2022_26591_MOESM1_ESM.docx]

**Supplemental Document 1**

Below are the details for the origin of each *Aedes albopictus* colony.

1. **New York**
   1. **Location:** Babylon, New York, USA (40.69, -73.33)
   2. **Collection method:** 22 ovitraps, 2 placed at each of 11 properties.
   3. **Dates of collection:** July 19 – 31, 2021
2. **Virginia**
   1. **Location:** Suffolk, Virginia, USA (36.73, -76.58)
   2. **Collection method:** 10 ovitraps, 1 placed at each of 10 properties
   3. **Dates of collection:** July 19 – July 29, 2021
3. **Maryland 1**
   1. **Location:** Harlem Park (39.29, -76.63) and Hollins Market/Union Square (39.28, -76.62) neighborhoods, Baltimore, Maryland, USA
   2. **Collection method:** Ovitraps; 1 ovitrap placed at each of 13 properties in Harlem Park and 1 ovitrap at each of 15 properties in Hollins Market/Union Square neighborhood. These neighborhoods were originally meant to be biological replicates, but due to low hatch rate, were combined into one colony
   3. **Dates of collection:** July 6 – 13, 2021
4. **Maryland 2**
   1. **Location:** Bolton Hill neighborhood, Baltimore, Maryland, USA (39.30, -76.62)
   2. **Collection method:** Larval collections; approximately 100 larvae were collected from around 20 containers spread over 5-6 city blocks.
   3. **Dates of collection:** August 31, 2021
5. **Florida**
   1. **Location:** Scrap yard, Vero Beach, Florida, USA (27.67, -80.43)
   2. **Collection method:** 10 ovitraps spread out within the scrapyard property
   3. **Dates of collection:** June 28 – July 26, 2021
6. **Cameroon 1**
   1. **Location:** Suburban neighborhood in Yaoundé, Cameroon
   2. **Collection method:** Larval collections from approximately 30 containers
7. **Cameroon 2**
   1. **Location:** Downtown Yaoundé, Cameroon
   2. **Collection method:** Larval collections from approximately 30 containers
8. **Thailand 1**
   1. **Location:** Ban Bueng District, Chon Buri, Thailand (13.263841, 101.136787)
   2. **Collection method:** 250 females were collected via human landing catch from 10-15 spots in vegetated areas, rubber tree plantations, and fruit orchards, with each spot about 20m or more apart.
9. **Thailand 2**
   1. **Location:** Pluak Daeng District, Rayong, Thailand (12.946102, 101.311641).
   2. **Collection method:** 151 females were collected via human landing catch from 10-15 spots in vegetated areas, rubber tree plantations, and fruit orchards, with each spot about 20m or more apart.
