# Supplementary material for: Aedes albopictus host odor preference does not drive observed variation in feeding patterns across field populations
Source: Sci Rep. 2023 Jan 4;13:130. doi: 10.1038/s41598-022-26591-3 (PMC9813369; doi:10.1038/s41598-022-26591-3)
Supplement: Supplementary file 2 — Supplementary Information 2. [file 41598_2022_26591_MOESM2_ESM.docx]

**Supplemental document 2**

We conducted a pilot comparison between two methods of human arm presentation, the elbow versus the forearm, to test whether a different method of presentation would increase attraction to a human who is deemed unattractive using the elbow presentation. This pilot was promising, showing markedly increased attraction of the Princeton anthropophilic *Ae. aegypti* to a human who attracted very few mosquitoes during the subject selection trials. We conducted a small trial to confirm this observation, testing both the Princeton anthropophilic *Ae. aegypti* and zoophilic *Ae. aegypti* colonies with both the forearm and elbow arm presentation methods. Three replicates were conducted for each replicate/presentation combination (Supplemental Figure 1A). The results were analyzed with a generalized linear mixed model using a betabinomial distribution, with group and human side as fixed effects. We confirmed that the forearm method of presentation indeed led to an increase in attraction of the anthropophilic *Ae. aegypti* to a relatively unattractive human compared with the elbow presentation (p = 0.0197, Supplemental Figure 1B). The zoophilic *Ae. aegypti* colony was also more attracted to the human with the forearm presentation method compared with elbow, but not significantly (p = 0.0650). Notably, the anthropophilic and zoophilic colonies did not have a significantly different probability of choosing human within each arm presentation type (forearm p=0.2175; elbow p=0.9777). This is in contrast to the significant differences that were found between these groups in both round one, round two, and combined model analyses. With only three replicates per group in this small experiment, there was not sufficient sample size to detect colony differences. However, that was not the goal of this trial, which was to show the difference in arm presentation method.

**Supplemental Fig1. a)** This scatter plot shows the proportion of mosquitoes that chose human over guinea pig for each group. Each point represents the results of one replicate, with color representing arm presentation method. Each colony and arm presentation combination is represented on the x axis; the “Anthro” colonies are the Princeton anthropophilic *Ae. aegypti* and the “Zoophil” colonies are the zoophilic *Ae. aegypti.* **b)** This graph shows the predicted probability of choosing human for each group (Tukey post-hoc analysis of GLMM, using glmmTMB and emmeans packages), with the bars showing the upper and lower confidence limits. The letters over each group represent statistical significance, with groups that do not share a letter having significantly different probabilities of choosing human (p<0.05)
