# Supplementary material for: Aedes albopictus host odor preference does not drive observed variation in feeding patterns across field populations
Source: Sci Rep. 2023 Jan 4;13:130. doi: 10.1038/s41598-022-26591-3 (PMC9813369; doi:10.1038/s41598-022-26591-3)
Supplement: Supplementary file 3 — Supplementary Information 3. [file 41598_2022_26591_MOESM3_ESM.docx]

**Supplemental table S1. Predicted probability of choosing human for each *Ae. albopictus* and *Ae. aegypti* colony based on generalized linear mixed models with data combined and for each experimental round separately**

| **Colony** | **Combined** | | | **First Round** | | | **Second Round** | | |
| --- | --- | --- | --- | --- | --- | --- | --- | --- | --- |
|  | **Probability** | **SE** | **Sig*** | **Probability** | **SE** | **Sig*** | **Probability** | **SE** | **Sig*** |
| *Ae. albopictus* |  |  |  |  |  |  |  |  |  |
| New York | 0.258 | 0.071 | A | 0.193 | 0.0803 | A | 0.346 | 0.0654 | A |
| Maryland 1 | 0.254 | 0.071 | A | 0.199 | 0.0779 | A | 0.314 | 0.0637 | A |
| Maryland 2 | —^‡^ | — | — | — | — | — | 0.293 | 0.0693 | A |
| Virginia | 0.304 | 0.0779 | A | 0.2051 | 0.0822 | A | 0.425 | 0.0691 | A |
| Florida | 0.306 | 0.0764 | A | 0.1961 | 0.0765 | A | 0.404 | 0.0709 | A |
| Cameroon 1 | 0.386 | 0.0866 | A | 0.2463 | 0.0976 | A | 0.557 | 0.0679 | AB |
| Cameroon 2 | — | — | — | — | — | — | 0.561 | 0.0699 | AB |
| Thailand 1 | 0.202 | 0.0672 | A | 0.2612 | 0.1075 | AB | 0.199 | 0.0597 | A |
| Thailand 2 | — | — | — | — | — | — | 0.338 | 0.0737 | A |
| *Ae. aegypti* ^ξ^ |  |  |  |  |  |  |  |  |  |
| Zoophilic | 0.206 | 0.0614 | A | 0.0385 | 0.0239 | A | 0.506 | 0.0751 | AB |
| Cornell anthropophilic | 0.735 | 0.0711 | B | 0.6408 | 0.2816 | BC | 0.818 | 0.0527 | BC |
| Princeton anthropophilic | 0.877 | 0.0432 | B | 0.7959 | 0.0785 | C | 0.957 | 0.0211 | C |
| Transport Control | — | — | — | — | — | — | 0.947 | 0.0248 | C |

*groups with different letters within a column represent colonies with significantly different probabilities of choosing human according to GLMM with Tukey post-hoc test, using glmmTMB and emmeans R packages.

^‡^colonies with dashes in the Combined and First Round columns were not assessed in the first experimental round and are therefore also not included in the combined model.
^ξ^zoophilic is a Ugandan colony, Cornell anthropophilic is a Thai colony established and reared at Cornell, Princeton anthropophilic is a Thai colony established and reared at Princeton, and Transport Control is the Princeton anthropophilic colony reared at Cornell.
